# Young carers in Germany: to live on as normal as possible – a grounded theory study

**DOI:** 10.1186/1472-6955-7-15

**Published:** 2008-12-24

**Authors:** Sabine Metzing-Blau, Wilfried Schnepp

**Affiliations:** 1 Institute of Nursing Science, Witten/Herdecke University, Stockumer Str. 12, 58453 Witten, Germany

## Abstract

**Background:**

In contrast to a growing body of research on the situation of adult family care givers, in Germany hardly anything is known about the situation of children and teenagers who are involved in the care of their relatives.

**Methods:**

In this Grounded Theory study 81 semi structured interviews have been carried out with children and their parents in 34 families, in which one member is chronically ill. 41 children and 41 parents participated and the sample is heterogeneous and diverse.

**Results:**

On the one hand, there is the phenomenon 'keeping the family together", which describes how families themselves cope with the chronic illness and also, which tasks to what extent are being shifted and redistributed within the family in order to manage daily life. Influencing factors, the children's motives as well as the impact on the children also belong to this phenomenon. The second phenomenon 'to live a normal course of life' describes concrete wishes and expectations of support for the family to manage the hindered daily life. These two phenomena linked together constitute the 'model of experience and construction of familial care, in which children take over an active role'.

**Conclusion:**

It will be discussed, that the more families are in dire need of support, the more their distress becomes invisible, furthermore, that management of chronic illness is a process, in which the entire family is involved, and thus needs to be considered, and finally, that young carer's relief is not possible without relief of their parents.

## Background

Research especially in the United Kingdom (UK) has led to a growing understanding of the situation of young carers over the last 15 years. According to these findings, the children are between eight and ten years old as they begin to take over caring tasks, and as young carers, they are on average 12 years old [1-3]. Girls as well as boys are involved [1-4], and more than half of them live in single parent families [1-3]. The parents in need of care are predominantly mothers with chronic somatic diseases.

According to a census from 2001 the prevalence of young carers in the UK is 1.5%, which in numbers are 175.000 children [5]. One has to be cautious with the transferability of data, but *if *the British prevalence rate were transferable to Germany, there would be more than 200.000 young carers in our country. These children, irrespective of their age, become involved in all areas of care and housekeeping [1-3,6-12]. The amount of their help varies and depends on many factors. Some assist the chronically ill just occasionally; others are solely responsible every day. The main influencing factors are the family's economic and emotional deprivation [13], its social environment [14], lack of outside support [7,13], specific circumstances such as the separation of parents [13] or the onset of the chronic illness [12,13], the process of being socialized into care [7,12,13] and, finally, the convenient availability of children at home [13,15]. Positive as well as negative effects can be found in the literature: positive ones are an increased sense of self-esteem, early maturity, a close relationship between the children and their parents as well as feeling well prepared for life [4,9,11-13,16-18]. Negative effects relate to physical, psychosocial and educational aspects.

Some children suffer from sleep disturbances and exhaustion, they feel lonely, feelings of sadness, fear and shame are mentioned frequently, other effects are loss of childhood, social isolation, as well as problems and missing time in school [1-3,11,13,16,18-23]. British researchers refer to the vulnerability of families concerned and they predict that children will be affected in their whole development if the families stay without support [13,24].

With a growing body of research on the situation of adult family caregivers both internationally and nationally, in Germany, hardly anything is known about the situation of children, who are involved in caring for their relatives. Thus we hardly know anything about their specific situations and their needs in our country. The study presented here intends to contribute to filling this gap.

The aim of this paper is to give an overview of the core findings, as the literature review and description of main concepts and categories have been published in more detail elsewhere [25-29].

## Methods

The aim of this study was to gain an insight into the situation of young carers and their families in order to provide a basis for the concept of family oriented support. The research questions concentrated on the construction of these familial caring situations as well as on influencing factors. We asked about kind and amount of care delivered by children, we were interested in the children's own experience of their situation, we wanted to learn about the impact on the children, and, with regard to the overall aim of the study, we asked for concrete wishes, needs and expectations towards support.

### Conceptual Framework

The study is based on a systemic approach. Chronic illness affects nearly all activities of daily life [30,31]. It not only affects the person in need, but also the surrounding social and, above all, the familial system [30,32,33]. Coping with chronic illness in the family cannot adequately be understood without a sensitive consideration of the specific kind of relationship between chronically ill persons and their significant others [31,32,34]. While our primary perspective is a systemic, family-oriented one, we also subscribe to a central position of modern childhood research which places the child at the very center of the attention [35,36] and addresses him or her as an expert of his/her world [36-38].

### Sampling and Recruitment

In order to adequately conceptualize the situation of young carers and to be able to come up with 'thick descriptions' of their situation, we aimed at a heterogeneous and diverse sample which would allow to include the multitude of relevant aspects of the phenomenon under concern and also to strengthen the credibility of the findings. While realizing that the final size would depend on data saturation, a sample size of about 30 families was striven. As expected, field access turned out to be extremely difficult and required multiple strategies for gaining access to appropriate families. We contacted over 200 health professionals^i^, 40 advisory boards and help desks^ii^, 50 self-help groups and 15 national self-help associations. Moreover, we repeatedly placed interview appeals in internet discussion forums, three press reports as well as a call-in radio show^iii^. In addition, we spread information material and placed an interview appeal in the member magazine of one of the country's biggest health insurance companies^iv^. In most cases, the families themselves contacted us. Quite frequently, it was the mothers who said that they wanted to give their children a chance to talk about their situation. This is important to keep in mind while interpreting the results, since the majority of this sample relies on self-identification. It is likely that children experiencing less parental support as well as more distress might report a higher degree of negative impact.

All in all, we carried out 82 interviews in 34 families in which one member suffered from a chronic illness (see Table 1). We interviewed families from all over Germany covering a variety of family constellations, social milieus, and numerous chronic illnesses. The interviews were heterogeneous, so that many relevant aspects of the phenomenon were represented. 16 families were single-parent families, predominantly with a single-parent mother. In the remaining 18 families, both parents lived in the same household (see Table 2). 41 children and 41 parents/grandparents (23 chronically ill, 18 healthy ones) participated (see Table 1 + 3). From all chronically ill persons, 21 of these chronic illnesses originated from a somatic account (Multiple Sclerosis, Stroke, Parkinson's Disease, Asthma, Cardiac Insufficiency, Cancer) 9 had a psychiatric background (Depression, Psychosis, Posttraumatic Disease), while four persons were somatically as well as a mentally ill (see Table 4).

**Table 1:** Crosstabulation: Interviewees * Ill person

		Ill person					
		Mother	Father	Grand-mother	Sibling	Parent + Grand-parent	Total

Interview with ill parent in the family	yes	16	5	1	0	1	**23**°
	
	no	7	2	0	0	0	9
	
	Total	23	7	1	2	1	32/34*

Interview with healthy parent in the family	does not exist	10	1	1	0	0	12
	
	yes	10	6	0	2	0	**18**°
	
	no	3	0	0	0	0	3
	
	Total	23	7	1	2	1	34

Interviews with daughters		16	7	0	1	1	25
		
Interviews with sons total		11	3	1	1	0	16
		
total		27	10	1	2	1	**41**°

* the total number of families is 34, missing 2: in two families the chronically ill person was a child

° marks the total number of n = 82 interviews carried out

**Table 2:** Crosstabulation: Ill person * Family constellation

		Family constellation			
		Single Parent	In Partnership	Parents + Grandparent	Total (%)
Ill person	Mother	13	10	0	23 (67.6)
	Father	1	6	0	7 (20.6)
	Grandmother	1	0	0	1 (2.9)
	Sibling	1	1	0	2 (5.9)
	Parents + Grandparent	0	0	1	1 (2.9)

	Total	16	17	1	34 (100)

**Table 3:** Overview: children's age cohorts and sex

		Sex		
		Girls	Boys	Total (%)
Age cohort	up to 7 years	5	1	6 (14.6)
	8 – 12 years	5	5	10 (24.4)
	12 – 15 years	12	7	19 (46.3)
	16 and older	3	3	6 (14.6)

	Total	25 (61)	16 (39)	41 (100)

children's age: minimum 4, maximum 19, mean 12,34

**Table 4:** Crosstabulation: Origin of illness * Ill person

		Ill person					
		Mother	Father	Grand-mother	Sibling	Parent + Grand-parent	Total
Origin of illness	Somatic	13	6	1	0	1	21
	Mental	8	1	0	0	0	9
	Somatic + Mental	2	0	0	2	0	4

	Total	23	7	1	2	1	34

^i ^Home care services, general practitioners and specialists, hospitals, medical professors from our own faculty who work in hospitals, cooperating partners of our Institute.

^ii ^Youth welfare offices, 'German Foundation for Child welfare (DKSB)', church help desks.

^iii ^*WDR5, LebensArt' 22.11.04 supra-region *how families master the multilayered situation concretely.

^iv ^Barmer Ersatzkasse; 02/2005, print run 6 million.

### Data Collection and Analysis

The data was collected over a period of 16 months; the place of the interview was chosen by the families. Before the interviews started, all participants received oral and written information about the study. Tape recording and anonymous transcription of the interviews were explained, and informed consent was obtained from every interviewee. While using semi-structured interviews, each interview was started with an open question. The code of practice for the interviews was conceived by using the results of a literature review [39] as well as the „Action Checklist“ provided by the UK's Department of Health [40], a small guideline specifically developed for interviewing young carers. We met with researchers from the, Young Carers Research Group' (University of Loughborough, GB) in order to discuss interview topics. All interviews showed a high degree of openness. The children were given the freedom to talk about issues that were relevant to them. Purposeful inquiries were restricted to the end of the interview session.

Data analysis was based on the method of Grounded Theory [41]. Due to the extremely difficult field access, almost every family that was willing to participate was included. Thus, in the beginning it was hardly possible to conduct theoretical sampling. Data analysis began as soon as the interviews were transcribed; this allowed for constant comparative analysis. As the study went on, we were able to do theoretical sampling in relation to our research questions.

### Ethical Approval

The study was approved by the Ethics Committee of the Institute of Nursing Science at the Witten/Herdecke University, Germany, as well as by the scientific review committee of the Federal Ministry for Education and Research.

## Results

**Figure 1:**
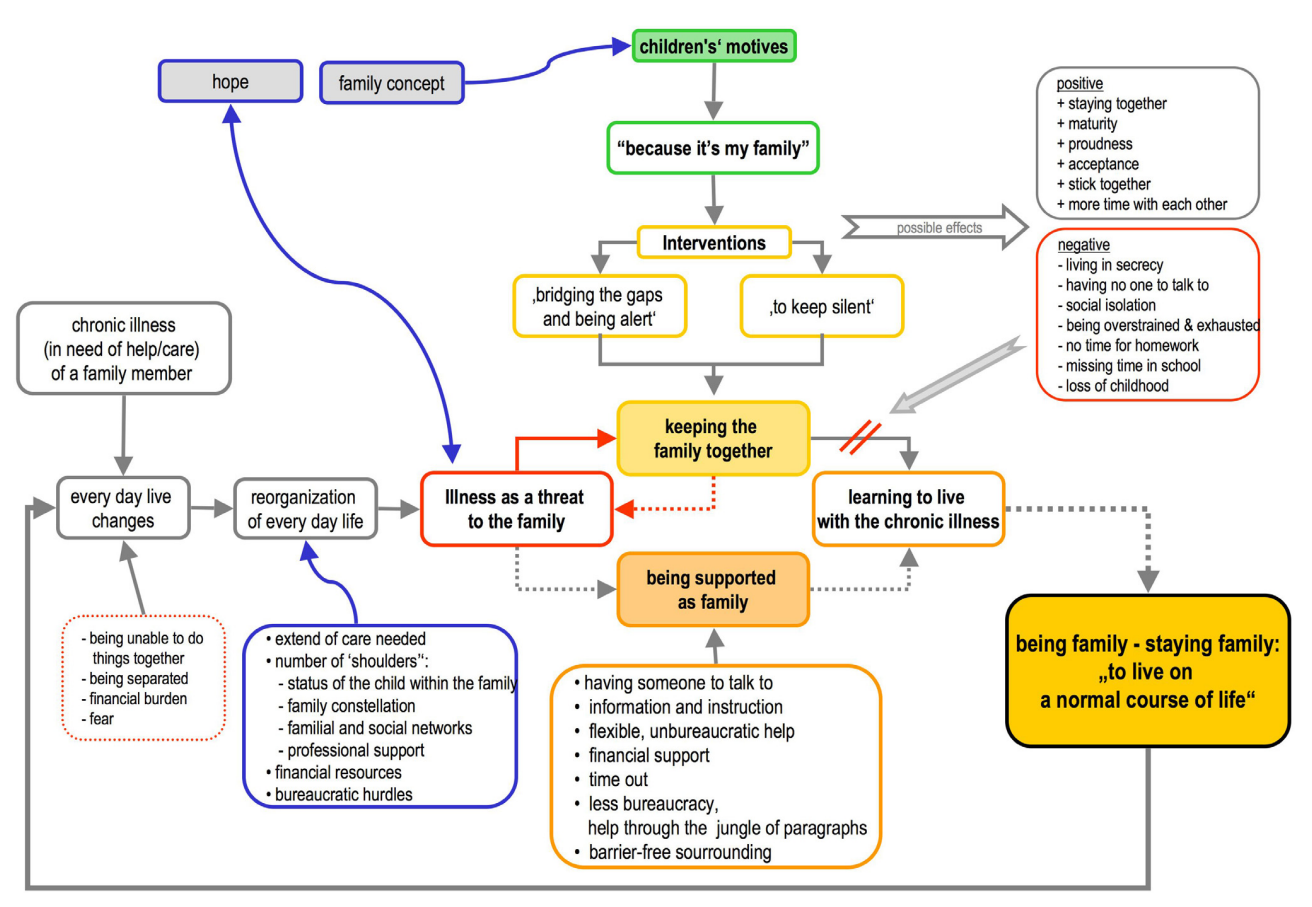
**Model of experience and construction of familial care, in which children take over an active role**.

Two central phenomena have been developed. On the one hand, there is the phenomenon: 'keeping the family together', which encompasses how families cope with the chronic illness and which tasks are being shifted and redistributed to manage every day life. Influencing factors, the children's motives as well as the impact on all family members, belong to this phenomenon. The second phenomenon: 'to live a normal course of life' describes the aspect of hope as well as concrete wishes and expectations towards outside support. These two phenomena when linked together constitute the 'model of experience and construction of familial care', in which children take over an active role (see Fig. 1) – which will be summed up in what follows.

Management of chronic illness affects the entire family, not only the chronically ill person. Every day life changes and must be reorganized. The children cannot do things with their parents as they used to, they also experience being separated from their parents due to hospital stays, and furthermore, financial shortages become an actual issue as well as a fear. At best, to anticipate the overall ambition of these families concerned, they manage to develop strategies that allow them to learn to live with the illness, and to live every day family life as normally as possible. Very often, this is not possible, because management of chronic illness is a dynamic process influenced by many factors.

### Influencing Factors

First of all, the severity of the illness and the necessity for care are the primary determinants of the necessary reorganization of every day life. Another influencing factor can be called the 'number of shoulders', which describes the number of people that are available to help with work. It makes a difference, whether we look at a single parent family without outside support or at a family where parents and maybe even grandparents and/or friends are available to help with work. Furthermore, financial shortages, the family's economic situation, as well as bureaucratic hurdles have influence. The more constrictive factors come together, the more the chronic illness dominates everyday life and becomes a threat to the family's normal course of life. Children worry about their parents and they fear to be separated as a family. The family now close ranks, and children take over an active part in the construction and maintenance of family life. Trying *to keep their family together*, they assume responsibility; their interventions are multilayered and complex.

### Bridging the gap and being alert

They 'bridge the gaps' that emerge, in fact regardless of the underlying illness (i.e. somatic or mental), and they put themselves on alert in order to react immediately to changes. Young carers do what adult informal caregivers do as well; they take over tasks which are left unaccomplished because of the illness. While in some cases such 'gap-bridging' may be provided just occasionally, in others adolescents may take on sole responsibility for caring tasks, and that "*around the clock*". To illustrate this, a 19 year-old girl reflecting on her time as a young carer will be quoted: *"I got up early in the morning and got ready myself, then I went to my Mum, helped her out of bed, washed her, dressed her, and then I put her in the wheelchair and went to school. After school I always had to run home, there was no time for a chat with my classmates, I always had to hurry up, because Mum needed to go to the toilet which she couldn't do without me. Thus it kept going on all day. Shopping, cooking and cleaning up. (...) But most of it was the care and the cleaning up*."

Since chronic illness is an unpredictable process, the children involved in such a situation will always be in a 'stand-by' and 'ready to act at any time' position beyond their regular tasks.

The concept 'keeping silence' also belongs to these interventions and it describes the children's secrecy. Hardly any of them talk to others about the situation at home, and this silence in most cases is rooted in shame and caution. Almost all of them mentioned that other children "*do not understand*", because they don't know what it means to cope with a chronic illness within the family. On the other hand, children usually want to go back to normality, they don't want to *"be different"*, and they are afraid of being isolated and excluded by their peers. Furthermore, you can observe a code of silence in these families, because many of them are in deep fear of being torn apart. From the children's perspective, intervention from the outside is often associated with separation, and a threat to the child's family also means a threat to the child's own identity as well.

### Motives for taking over caring tasks

Even though, many children deliver care to a great extent, for most of these young people, without question, their help is a matter of course, and this may be understood by looking at their motives for taking over caring responsibility. These did not belong to the research questions in the first place, but as the inductive analysis went on, children's motives seemed to play an important role in understanding families' strength. The children are socialized into their caring tasks, and as the chronic illness progresses, kind and extent of their tasks will change and increase. This adaptation, often hardly recognized, does not occur as a consequence of a conscious decision-making process in favor of becoming a carer. Caring tasks arise and change in the course of the chronic illness. For the youngest (preschool- and elementary schoolchildren), the wish to help "*Mummy*" dominates over everything else, and they do not experience their help as a burden. As age increases, the children's awareness of the necessity for their help also rises. For these children, the awareness of being responsible resides over the pride of being able to make a contribution. They know that their family relies on them. A 14 year-old girl said: "*my siblings are little, I can not say 'I can't stand it any longer', (...)' I must be there for them, that's where the power comes from*.' and she adds: "*because it's my family, (...) I want to be there for my family*"'. Finally, looking at adolescents, their motives are similar to those of the younger children; this particularly holds true for the motive of affection for the ill parent. However, feeling responsible for the family and moving together as a family not only emerges from the acute situation and from sheer necessity. Adolescents explain their caring also by referring to their emerging sense of morality. A 17 year-old girl said: *"I would never forgive myself if I wouldn't do it"*, and an 18 year-old boy reasoned: '*that's not what I learned when growing up, to walk out on someone and say: 'just do your shit alone'*'.

### Impact on the children and the family

There is no doubt, that the various caring activities do have implications on the children's development – positive as well as negative ones. In accordance with British research results (see page 3), positive effects as mentioned by young carers are their increased sense of self-esteem, the feeling of being *"one step ahead"*, early maturity, as well as a strong sense of coherence. They are important resources that need to be considered when thinking of support. We would like to focus on the negative effects, because they are the cause for concern. These include the secrecy of the family's distress, and the fact, that young carers usually have no one to talk to. Social isolation has to be mentioned, because many young carers have no time left for friends; a 14 year-old girl, oldest child of a badly traumatized mother, told us that it makes her sad to see all other children playing outside except for her. Furthermore, we also observed difficulties in school and even poor school attendance.

If families are left alone with their situation, and if children are overstretched with their caring responsibility, the families are in danger. They will try to uphold every day life, but a normal course of life is out of reach; this leads to a disruption in the process. If the illness remains a threat for the family, a vicious circle begins. The families totally close their ranks, some even isolate themselves. The children intensify their activities; everything is subordinated to the management of every day life; protection of the family dominates over everything. Negative effects of caring on the children's development will increase.

### Family oriented support

Many families experience a lack of adequate support from outside, and they often don't even know of existing support services due to a lack of information. Professional advice coordinated by one central contact-point does not exist. For most children and their parents, emotional support has first priority. They want 'someone to talk to', professionals as well as peers. Furthermore, they expect and claim flexible and unbureaucratic help, which considers and respects their reality. A chronically ill mother said: *"one phone number and I'd know, I am in good hands, they will help me and won't come with paragraphs and thousands of application forms. Within two hours I would get something with rhyme and reason, and I'd know how to move along." *If – for example – a chronically ill parent wakes up with an acute attack of Rheumatism or Multiple Sclerosis, unable to get up, he or she wishes to be able to call one central number where low-threshold help will be organized. In this example, help could mean to have someone come over and pick up the children for kindergarten or school. Additionally, it may also mean that someone will help with cooking and cleaning that day, and a nurse may come and look after the parent. Many children also ask for age-based information and education about the illness and about the care needed. They want to understand and be able to help more sufficiently. A 14 year-old boy described, that his mentally ill (and single parent) mother sometimes is *"different"*, he said: *"then she cries and screams, she stands in the room and gazes at one spot for hours, and she cries and all that, and so it goes on and on." *His reaction towards such situations is affected by uncertainty. The doctors, he said, always prescribe pills, but they don't tell him how to respond to help his mother. For children, whose parents are disabled, practical nursing skills stand in the foreground. Further needs are help with paragraphs and application forms as well as time out. The family's own input, combined with externally provided evidence based, family oriented support, determined by the family's specific needs, will help to overcome this vicious circle and support families in their effort to live in the way they wish to, despite chronic illness. Above all this, we need to take into account, that management of chronic illness is a dynamic process, and any change occurring in the course of the illness as well as in the family itself demands the reorganization of every day life.

## Discussion

The findings of this study confirm once more that management of chronic illness involves the entire family and thus the entire family needs to be considered. Children can not and should not be shielded against parental illness. The illness belongs to the family's life, and children will always try to make their contribution to the management of everyday life. But there are capacity limits, and if these are touched and even exceeded, there is an urgent need for support. For most of the families it is important to organize every day life with the illness – without letting it dominate their life. The family stands in the foreground, not the chronic illness. But chronically ill people – despite an established social and health care system – are hardly being perceived as part of a social and familial system in the German health care system.

The German health care system dates back to 1883, when health insurance was nationwide made mandatory for certain employees ("statutory health insurance") by law [37]. The German social insurance system was extended through the work-related accident insurance (1884), the old-age pension insurance (1889), the unemployment insurance (1927) and the long-term care insurance (1995). The long-term care insurance consists of two parts: the mandatory social long-term care insurance and the mandatory private long-term care insurance [[37], p. 116]. "Benefits are available upon application only. The Medical Review Boards (...) evaluate the applicants and place them into one of the three categories (or deny care). (...) Beneficiaries with a care dependency then have a choice of receiving monetary benefits or professional nursing care while staying at home or to receive professional nursing services in nursing homes. The benefits of long-term care insurance are graded according to type, frequency and duration of the need for nursing care. (...) Monetary support is intended to cover home care delivered by family members (...) plus a professional substitute (...) to cover holidays. (...) In addition, family members serving as care-givers at home can attend training courses free of charge, and short-term care is provided during holidays of care-givers. The care-giver is also covered by statutory accident insurance and statutory retirement insurance, financed by the sickness fund administering the long-term care insurance of the person in need" [[37], p.117f].

As long as treatment plans ignore a persons real world, and as long as they disregard a) the meaning, which the chronic illness has for the person as well as b) how a chronically ill person wants to live everyday life with and within the family, these people will not follow the treatment plans but make their own decisions, trying to find compromises that they can bear. Nevertheless, they run the risk of being more restricted through their illness as would be necessary with adequate patient and family oriented treatment. Furthermore, children serving as care-givers can not benefit from the long-term care insurance as informal adult care-givers can. There are, for example, no age-based training courses for children and adolescents available.

The aim of the study was to gain insight into the situation of young carers and their families in order to build up a basis for family oriented support. Not all of the described influencing factors can be changed or controlled. No supportive intervention can influence the severity of the illness or the need for care. But it can influence, how a family manages the situation and what resources are given to the family, the chronically ill person as well as to the care-giver, especially to the children. As described in the 'model of experience and construction of familial care, in which children take over an active role', supportive interventions need to consider the complexity of the situation.

The ongoing and current research project achieves the aim to develop, implement and evaluate an evidence based intervention to support young carers and their families in the management of their hindered normal course of life. The concept for this intervention is based on the research findings described in this manuscript as well as on expert interviews, which have been carried out with leaders of young carers' projects in the UK. Until recently, most of the British projects focus on young carers but not on the family as a whole. But young carer's relief will not be possible without relief of their parents. The family's well-being has first priority for the children. They themselves point out, that the entire family is affected by the chronic illness, thus everyone in the family should be able to participate in supportive interventions.

Furthermore how parents cope with their chronic illness has great influence on a child's experience. Many study results show, that the way parents handle their illness has greater influence on the children's experience, on symptoms of fear and depression than the severity of an illness [42-45]. Thus, to relieve young carers, it is essential to also support parents in the individual process of managing their illness. The concept for this intervention has already been developed, the implementation process will start in 2009, first results of this trial (registration number: NCT00734942) can be expected in the beginning of 2010, results of the preliminary literature review have been published elsewhere [46].

## Conclusion

The findings of this study confirm many of the British research results and they give an insight into the situation of young carers and their families in Germany. The 'model of experience and construction of familial care' contributes to the body of knowledge on young carers as it helps to understand the relationship between categories, and it therefore may help to assess a family's situation according to influencing factors, strengths' and resources as well as burden and impact on the children. Families are master craftsmen in the management of everyday live. Not every child that grows up with a chronically ill parent will be afflicted with this situation, nor will it automatically become a young carer. But those, who are overstretched with the situation, need family oriented support. One finding of this study may suit as a tenor for such an intervention in order to not interfere more than necessary and wanted: „bridging the gap and being alert“.

## Abbreviations

UK: United Kingdom

## Competing interests

The authors declare that they have no competing interests.

## Authors' contributions

SMB and WS were responsible for defining the research questions, WS for the drafting of the study proposal. SMB was responsible for the drafting of this paper, which is a summary of her doctoral dissertation. WS read and approved the final version.

## Pre-publication history

The pre-publication history for this paper can be accessed here:


